# Testing Initiatives Increase Rates of HIV Diagnosis in Primary Care and Community Settings: An Observational Single-Centre Cohort Study

**DOI:** 10.1371/journal.pone.0124394

**Published:** 2015-04-17

**Authors:** Prini Mahendran, Suneeta Soni, Stephanie Goubet, Emma Saunsbury, Jonathan Roberts, Martin Fisher

**Affiliations:** 1 Department of GU Medicine, Brighton and Sussex University Hospitals NHS Trust, Brighton, United Kingdom; 2 Clinical Investigations and Research Unit, Brighton and Sussex University Hospitals NHS Trust, Brighton, United Kingdom; 3 Brighton and Sussex Medical School, Brighton, United Kingdom; Alberta Provincial Laboratory for Public Health/ University of Alberta, CANADA

## Abstract

**Objectives:**

The primary objective was to examine trends in new HIV diagnoses in a UK area of high HIV prevalence between 2000 and 2012 with respect to site of diagnosis and stage of HIV infection.

**Design:**

Single-centre observational cohort study.

**Setting:**

An outpatient HIV department in a secondary care UK hospital.

**Participants:**

1359 HIV-infected adults.

**Main Outcome Measures:**

Demographic information (age, gender, ethnicity, and sexual orientation), site of initial HIV diagnosis (Routine settings such as HIV/GUM clinics versus Non-Routine settings such as primary care and community venues), stage of HIV infection, CD4 count and seroconversion symptoms were collated for each participant.

**Results:**

There was a significant increase in the proportion of new HIV diagnoses made in Non-Routine settings (from 27.0% in 2000 to 58.8% in 2012; p<0.001). Overall there was a decrease in the rate of late diagnosis from 50.7% to 32.9% (p=0.001). Diagnosis of recent infection increased from 23.0% to 47.1% (p=0.001). Of those with recent infection, significantly more patients were likely to report symptoms consistent with a seroconversion illness over the 13 years (17.6% to 65.0%; p<0.001).

**Conclusions:**

This is the first study, we believe, to demonstrate significant improvements in HIV diagnosis and a shift in diagnosis of HIV from HIV/GUM settings to primary practice and community settings due to multiple initiatives.

## Introduction

In 2011 there were an estimated 96,000 individuals in the UK infected with human immunodeficiency virus (HIV), of whom 24% were undiagnosed [1]. 47% of individuals were diagnosed late—with a CD4 count of less than 350 cells/mm^**3**^ (the European consensus definition of late presentation [2]); such late diagnosis is associated with a significant increase in mortality [3]. Undiagnosed infection is associated with greater risk of onward transmission [4], and late diagnosis with increased healthcare costs [5].

In order to reduce rates of undiagnosed HIV, the UK and other countries have introduced multiple strategies to broaden HIV testing. British guidelines recommend routine testing in acute general medicine and primary care in areas where local HIV prevalence is greater than 2/1000, and both the UK and European guidelines recommend the routine offer of a HIV test for patients presenting with a clinical indicator disease [6,7]. Studies have shown that HIV-infected individuals who test late have often presented to healthcare facilities on multiple occasions within the last 2 years prior to their diagnosis, where the opportunity for a more timely diagnosis has been missed [8].

It is increasingly recognised that older persons (usually defined as >50 years of age in the setting of HIV infection) are a significant group affected by HIV: an increasing number of older individuals are acquiring HIV, older individuals have more rapid progression of HIV infection, and immunological response to antiretroviral therapy (ART) may be suboptimal [9]. Current testing initiatives are less effective in reducing late diagnosis rates in such older persons [10].

Brighton has a high prevalence of HIV (7.59/1000 2011 [1]) and the largest single-centre cohort of HIV-positive individuals in the UK outside London. Over the past 13 years, Brighton has been an early implementer of newer approaches to HIV testing including routine testing in antenatal care and genitourinary medicine (GUM). Brighton was also chosen by the Department of Health as a pilot site for both community-based point of care testing, routine testing in acute general medicine and primary care, and has initiated enhanced testing initiatives for patients who present with clinical indicator diseases.

## Objectives

The main objectives of this study were:

- To examine trends over time of all new HIV diagnoses made in Brighton and Hove between 2000 and 2012 with respect to site of diagnosis and stage of HIV infection.
- To measure any association with age

## Materials and Methods

### Design

An observational single centre cohort study.

### Subjects

Subjects were adult patients receiving their initial HIV diagnosis in Brighton and Hove between 2000 and 2012.

### Setting

An outpatient specialist HIV department in secondary care.

Information was collated which had already been routinely collected as part of surveillance from patient notes, the HIV clinic database and the laboratory results reporting system. Data collated included site of initial HIV diagnosis, stage of HIV infection, age, gender, ethnicity, and sexual orientation.

Subjects were included if they received their first documented HIV positive antibody result and were seen for care in the HIV department at the Royal Sussex County Hospital in Brighton between 2000 and 2012. Subjects were excluded if they had received care for HIV infection in any other HIV healthcare setting prior to attendance at this treatment centre.

Testing in community sites and in the primary care pilot project used the Determine HIV-1/2 (Abbott Laboratories) point-of-care test (POCT). All those who tested positive by POCT required confirmation by 4^th^ generation venous blood testing. Community testing sites targeting men who have sex with men included specific questioning of recent risk behaviour and/or symptoms consistent with HIV seroconversion. If either of these were present and the POCT was negative, individuals were referred on to GUM services for repeat testing with a 4^th^ generation test, as above.

Individuals were classified as being diagnosed late if their first CD4 count at HIV diagnosis was less than 350 cells/mm^3^, and with advanced disease if their initial CD4 count was less than 200 cells/mm^3^ (consistent with the European Consensus definition of late HIV diagnosis [2]). Individuals were classified as being diagnosed with recent HIV infection if they fulfilled the criteria used by the Medical Research Council register of HIV seroconverters i.e. one or more of: previous negative antibody test within 1 year, positive HIV antigen test and simultaneous negative antibody test, or incident infection as defined by the Recent Infection Testing Algorithm (RITA) [11]. For those patients diagnosed with recent infection, information on symptoms consistent with seroconversion were collected at their initial consultation [12]. The identification of recent HIV infection/seroconversion was considered important as multiple studies have suggested that this is a time of significantly enhanced risk of HIV transmission [13] and that the opportunity for diagnosis is frequently missed [14].

Site of diagnosis was grouped according to those sites where testing has been recommended nationally throughout the study period (Routine settings such as genitourinary medicine, antenatal care, blood transfusion), and those sites where newer initiatives have recommended testing (Non-Routine settings such as primary care, community settings, medical outpatients, and medical inpatients).

Our underlying hypothesis for performing this study was that by expanding HIV testing to Non-Routine settings we would see a shift in the site of new HIV diagnoses.

### Ethical approval

Prior to commencing the study we checked using the Health Research Authority / Medical Research Council decision tool [15] according to which it was deemed unnecessary to gain ethics committee approval for this type of project which did not require randomization, did not require changing patient treatment, and utilized anonymous patient data that had already been collected routinely as required for routine surveillance. Written informed consent was not given by participants for their clinical records to be used in this study. Patient data was anonymized and de-identified prior to analysis.

### Statistical analysis

The data was analysed using SPSS (Statistical Package for the Social Sciences) 20. Proportions were compared using chi-square or Fisher’s exact test. Trends over time were analysed using Mann-Whitney test.

## Results

Between 2000 and 2012, 1359 adult patients were newly diagnosed and seen for care in this HIV department. The median age was 35 years (IQR 29–43); 85.0% were male, 77.1% were white, and 87.0% of men identified themselves as men who have sex with men.

**Fig 1:**
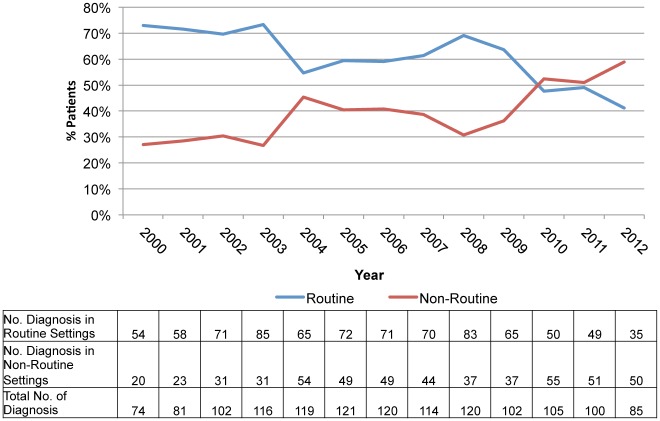
HIV diagnosis by location over time. A significant increase was seen in the proportion of new HIV diagnoses being made in Non-Routine settings.

**Fig 2:**
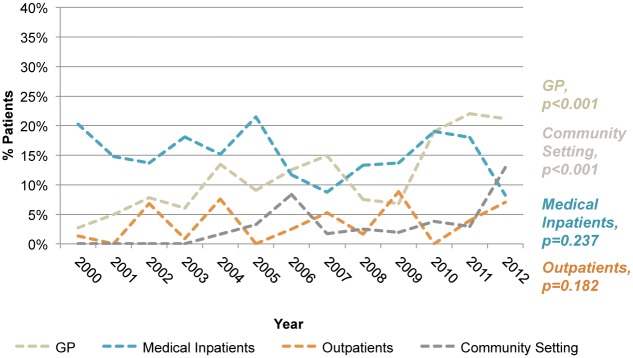
Non-Routine HIV diagnosis by location over time. Within the Non-Routine settings, a steady and significant increase in diagnoses was made within primary care and community settings.

The location of diagnosis data is shown in Figs 1 and 2. This demonstrates a significant increase in the proportion of new HIV diagnoses being made in Non-Routine settings (from 27.0% in 2000 to 58.8% in 2012; p<0.001), in particular, a steady and significant increase in diagnoses was made within primary care (2.7% to 21.2%; p<0.001). There was no significant change in the proportion of new diagnoses made in either medical inpatients or outpatients during the study time period.

Overall there was a decrease in the rate of late diagnosis from 50.7% to 32.9% (p = 0.001); this observation was made in both Non-Routine settings from 89.5% to 42.0% (p<0.001) and Routine settings from 37.0% to 20.0% (p = 0.018).

Recent infection increased from 23.0% to 47.1% (p = 0.001) during the study period; in Non-Routine settings this increased from 15.0% to 40.0% (p<0.001) and in Routine settings from 25.9% to 57.1% (p = 0.003). Of those with recent infection, over time significantly more patients were likely to report symptoms consistent with a seroconversion illness (17.6% to 65.0%; p<0.001).

Of those individuals aged 50 years or over at the time of initial diagnosis (n = 159), the site of diagnosis was primarily in Non-Routine settings and indeed increased within Non-Routine settings from 55.6% to 100% (p<0.001) over the study period.

## Discussion

A number of novel testing initiatives have led to a significant change in the site of new HIV diagnoses, an increasing proportion of which are now made in Non-Routine settings. We have shown that multiple initiatives in primary care and community settings can improve the recognition and diagnosis of HIV. In Brighton this has been achieved through the following:

- a designated HIV testing liaison health advisor post. This post was funded to assist non-GUM physicians in primary and secondary care with the offering of HIV testing, results giving, post-test discussion and linkage into specialist HIV care
- a specific HIV educational course aimed at primary care physicians and nurses including training around recognition of indicator diseases
- specific work with individual specialties to increase recognition of indicator diseases
- simplification of testing processes to enable non-GUM physicians to offer a brief and effective pre-test discussion and offer testing as part of a routine consultation
- co-working with community-based organisations to improve testing in outreach settings, for example rapid HIV testing in a community-based headquarters and sex on premises venues.

Encouragingly, our data demonstrates that it is possible to improve the detection of recent HIV infection, which should have a significant impact on the risk of onward HIV transmission from a public health perspective. The data also suggests that seroconversion symptoms are more likely to be reported by patients over time and also that the increase in new diagnoses of recent infection are primarily in Non-Routine settings, supporting the need for a strong and sustained educational message to support both patients and clinicians in recognising the symptoms and signs of early HIV infection.

During the study period, POCTs for HIV were used uniformly in all community settings and during the initial six-month pilot of testing in primary care, as well as in specific clinical scenarios where an immediate test result was required, for example if a female of unknown antenatal HIV status presented in labour. During the study period, 43 new diagnoses were made using POCTs (41 in community settings, and 2 in primary care). The requirement for immediate linkage to care and confirmatory testing in all individuals who had a reactive POCT ensured that there were no “false positive” HIV diagnoses given to patients. Testing programmes that adopt POCTs for HIV need to take into account the risk of false reactive tests, particularly when used in a low prevalence setting [16]. Conversely, recommending that individuals reporting recent high-risk behaviour or seroconversion symptoms undergo additional or repeat testing if the initial POCT is non-reactive, reduced the risk of missing acute infection. This is of particular importance given the disproportionate contribution of these individuals to onward transmission [17]. Our findings therefore suggest that the addition of POCT in settings where venepuncture may otherwise inhibit testing can form part of a programme to enhance HIV testing, as long as the caveats of false reactive tests and the potential for missed primary infection are integral to the care pathway.

Including data from this cohort, it is increasingly recognized that HIV is affecting older individuals, that older individuals are increasingly being newly infected with HIV and that they are disproportionately diagnosed late [1,10]. This study also reports that such individuals are far less likely to present to Routine settings and therefore Non-Routine settings, particularly primary care and non-specialist secondary care, need to be aware of the possibility of undiagnosed HIV infection in older persons. Clinical indicator diseases, as listed by the British and European guidelines [6,7], must be an indication for testing irrespective of age.

Despite these efforts, late diagnosis continues to be a significant issue. This is of major concern given that much HIV-related morbidity and mortality is associated with late diagnosis. We have demonstrated that improvements in testing have occurred in primary care and community settings but there has been little or no change in secondary care. Given that many individuals presenting with a late HIV diagnosis are known to have presented to secondary care in the recent past, there remains a significant challenge in engaging and educating non-HIV clinicians to recognise HIV indicator diseases and in normalising the process of HIV testing. It is of note that all of the recent HIV testing pilot studies have shown that patient acceptability towards testing has been extremely high and that the major barrier to routine testing has been the reluctance of clinicians to offer the test [18].

The strengths of our data are that it is from a high prevalence area, where HIV testing and clinical management are from one unit, the data is complete, patient follow-up is confirmed, and that demographic and clinical data were collected prospectively. The limitations are that our cohort is not entirely representative of that within the UK, being predominantly men who have sex with men, although this is the major group where onward transmission is occurring.

## Recommendations

A multifaceted and multidisciplinary approach to HIV testing can significantly increase HIV testing in Non-Routine settings.

A combination of patient education and clinician education can improve the recognition of primary HIV infection, which may have a significant impact on the HIV epidemic.

Recognition of HIV clinical indicator diseases should be a routine component of undergraduate and postgraduate general medical training.

The process of HIV testing and the informed consent process needs to be re-addressed in recognition of current testing guidelines and GMC guidance.
